# Is adjuvant chemotherapy necessary for young women with early-stage epithelial ovarian cancer who have undergone fertility-sparing surgery?: a multicenter retrospective analysis

**DOI:** 10.1186/s12905-022-01642-z

**Published:** 2022-03-21

**Authors:** Yoshiki Ikeda, Masato Yoshihara, Nobuhisa Yoshikawa, Akira Yokoi, Satoshi Tamauchi, Kimihiro Nishino, Kaoru Niimi, Hiroaki Kajiyama

**Affiliations:** Department of Obstetrics and Gynecology, Nagoya University Graduate School of Medicine, Truma-cho 65, Showa-ku, Nagoya, Aichi 466-8550 Japan

**Keywords:** Fertility, Carcinoma, Ovarian epithelial, Drug therapy, Propensity score

## Abstract

**Objective:**

In young patients with early-stage epithelial ovarian carcinoma (EOC) who were received fertility-sparing surgery (FSS), the role of adjuvant chemotherapy is unclear. Here, we performed a multicenter study using inverse probability of treatment weighting (IPTW) to explore the effect of chemotherapy on patients’ survival.

**Methods:**

Between 1987 and 2015, a retrospective study was carried out, including 1183 patients with stage I EOC. Among them, a total of 101 women with stage I EOC who underwent FSS were investigated, including 64 and 37 patients with or without adjuvant chemotherapy, respectively. Oncologic outcomes were compared between the two arms using original and IPTW cohorts.

**Results:**

During 62.6 months (median) of follow-up, recurrence was noted in 11 (17.2%) women in the chemotherapy arm and 6 (16.2%) patients in the observation arm. In the unweighted cohort, the 5-year overall and recurrence-free survival (OS/RFS) rates of chemotherapy and observation arms were 86.3/80.8 and 90.2/79.8%, respectively. There was no significant difference between the two groups {Log-rank: *P* = 0.649 (OS)/*P* = 0.894 (RFS)}. In the IPTW cohort after adjusting for various clinicopathologic covariates, we also failed to identify a difference in RFS/OS between the two groups {RFS (chemotherapy vs. observation), HR: 0.501 (95% CI 0.234–1.072), *P* = 0.075: OS (chemotherapy vs. observation), HR: 0.939 (95% CI 0.330–2.669), *P* = 0.905}.

**Conclusions:**

Even after adjusting clinicopathologic covariates, performing adjuvant chemotherapy may not improve the oncologic outcome in young patients who have undergone FSS.

**Supplementary Information:**

The online version contains supplementary material available at 10.1186/s12905-022-01642-z.

## Introduction

Epithelial ovarian cancer (EOC) is the one of the most aggressive malignancies in the female genital tract, with more than 22,530 newly diagnosed patients and 13,980 deaths/year in the United States [1]. EOC is common in postmenopausal women. In fact, according to earlier reports, approximately 10% of women with this carcinoma are under or around 40 years of age [2, 3]. Certainly, what is most important for patients and surgeons is to seek the complete cure of EOC. However, if we adopt radical surgery, such as hysterectomy, bilateral salpingo-oophorectomy, and various surgical staging operations for reproductive-age patients, the possibility of child-bearing as well as endocrine function will be lost. Preserving such female-specific fertility is critical to maintain patients’ quality of life. Therefore, fertility-sparing surgery (FSS), consisting of at least conservation of the uterus and contralateral ovary, is normally applied for those patients with early-stage EOC. A desirable candidate for FSS is typically a patient with a well-differentiated/ovarian-confined EOC [4]. However, we sometimes encounter a clinical situation where we perform FSS for women with capsule-ruptured, poor-differentiated EOC, and positive ascites if they accept the risk of recurrence. In clinical practice, we expect chemotherapy to eradicate occult metastasis. Actually, patients who undergo FSS frequently receive adjuvant chemotherapy to prevent unexpected recurrence in the future. Currently, the administration of postoperative chemotherapy is recommended for early-stage EOC women with a high recurrence risk, including stage IA/clear-cell type or stage IC/non-clear-cell histology [5]. On the other hand, in optimally staged patients, earlier European studies failed to show a significant difference in overall survival between chemotherapy and observation arms [6]. A critical and controversial issue after chemotherapy is ovarian toxicity, one of the major side-effects affecting young women with malignancies. Indeed, it is controversial whether such women benefit from postoperative chemotherapy.

To date, a propensity score technique has been utilized to minimize or exclude the effects of a number of confounders in observational studies. In particular, a statistical methodology of inverse probability of treatment weighting (IPTW) has been reported to contribute to better adjustment for measurable and unmeasurable confounders in order to minimize selection bias. Here, we explored the impact of postoperative chemotherapy on oncologic outcomes in young women with early-stage EOC who received FSS in a multicenter analysis using IPTW methodology.

## Methods

### Patient enrollment

Between 1987 and 2015, a retrospective, observational cohort study was carried out analyzing 1183 women with stage I EOC based on a search of the medical documents from 14 collaborating hospitals. All histological slides were reviewed by expert pathologists with no knowledge of the patients’ clinical data under a central pathological review system. Borderline tumors were excluded in this study. Among them, further eligible cases were extracted according to following criteria: (1) histologically confirmed stage I EOC, (2) aged under 45 years old at the time of initial diagnosis, (3) underwent initial FSS, and (4) received periodic follow-up in each institution. Consequently, 101 women with stage I EOC who had undergone FSS, were collected. Histological types were pathologically diagnosed according to criteria of the World Health Organization (WHO) classification. The stage was assigned based on the classification of International Federation of Gynecology and Obstetrics (FIGO) [7, 8]. The current study was conducted as a secondary analysis following our recent study investigating oncologic outcomes between FSS and radical surgery cohorts [9]. The present study was approved by the Ethics Committee of Nagoya University according to the principles of the Declaration of Helsinki.

### Treatments

FSS was defined as at least conservation of the uterus and contralateral ovary. Women who received FSS had a strong desire to preserve their fertility, and were informed of the possible risks and benefits of FSS in a preoperative counseling session with written consent forms. Omentectomy and wedge resection of the contralateral ovary were optional. Lymph node evaluation involved one of the following: (1) careful palpation and removal of enlarged lymph nodes (larger than 1 cm in diameter), (2) lymph node sampling, or (3) lymph node dissection [9]. Overall, 64 (63.4%) were received postoperatively with 3–6 cycles of adjuvant platinum-based chemotherapy. A total of 37 (36.6%) women did not undergo adjuvant chemotherapy due to the decision of each institution, including the patients’ hope and criterion of omission (e.g., stage IA/grade 1–2). Details of the chemotherapy regimen in our group were previously described [10].

### Follow-up and analysis

All patients principally received a careful follow-up, including a gynecologic examination, CA125 evaluation, ultrasonographic scan, and periodic radiologic imaging based on the Gynecologic Cancer InterGroup (GCIG) criteria [11]. The recurrence-free survival (RFS) was defined as the time interval between the date of surgery and that of recurrence or the last follow-up. The overall survival (OS) was defined as the time between the date of surgery and that of the last follow-up or death from any cause. To balance the covariates between chemotherapy and observation arms, the propensity score (PS)–weighting technique was adopted [12]. PS was calculated by multivariable logistic regression models for the probability of chemotherapy performance, adjusting for age (> 35 vs. ≤ 35 years), FIGO stage (IC2/IC3 vs. IA/IC1), histological type (non-mucinous vs. mucinous), preoperative CA125 value (> 35 vs. ≤ 35 U/mL), volume of ascites (> 100 vs. ≤ 100 mL), and cytology of ascites (positive vs. negative). Women were weighted based on the inverse probability of receiving chemotherapy according to the previously-reported method [13]. Using this statistical technique, each case was weighted by the inverse probability of being in the chemotherapy versus observation group, aiming to balance the observed characteristics between the two cohorts. Within the original (unweighted) and weighted cohorts, survival curves were generated using Kaplan–Meier methods. A Cox proportional hazards regression model was employed to examine multivariable analyses. The distributions of clinicopathologic events were evaluated using the Chi-squared tests or Student *t*-test. All statistical analyses were carried out with SPSS Ver. 26 (IBM Japan, Tokyo) and JMP Pro Ver.10.0 (SAS Institute Japan). A *P* value of < 0.05 was considered significant.

## Results

### Clinicopathologic characteristics

Patients’ characteristics are presented in Table 1. Overall, there were 64 (63.4%) who received chemotherapy and 37 women (36.7%) who did not receive chemotherapy. The median (SD) age of patients with or without chemotherapy was 34 (7.2) years and 31 (8.1), respectively (*P* = 0.160). The median follow-up duration of all women was 62.6 months. With regard to the FIGO stage, a stage IC tumor was more commonly observed in the chemotherapy arm than in the observation arm (*P* = 0.0424). Additional file 3: Table S1 shows the distribution of the performance of chemotherapy in each substage/histological type. In particular, among 15 patients with stage IC2/IC3 tumor, the majority of cases received chemotherapy {86.7%: (N = 13)}. Additionally, the preoperative CA125 value was higher in the chemotherapy arm than in the observation arm (*P* = 0.008). With regard to the distribution of the ascites cytology, histological types, and, volume of ascites, no difference was identified between the two arms. Multivariable analysis showed that a higher preoperative CA125 value (> 35 vs. ≤ 35 U/mL: *P* = 0.030) and substage (IA vs. IC: *P* = 0.049) were significantly correlated with the performance of chemotherapy (Additional file 4: Table S2).

**Table 1:** Patients' characteristics

	Total	Chemotherapy		Observation		*P* value*
		N	%	N	%	
Total	101	64		37		
Age (median/mean/SD)		34/32.8/7.2	31/30.6/8.1	0.160		
≤ 35 years	58	34		24		
> 35 years	43	30		13		
FIGO stage						
IA	43	21	32.8	22	59.5	0.0424
IC1	43	30	46.9	13	35.1	
IC2	8	7	10.9	1	2.7	
IC3	7	6	9.4	1	2.7	
Histological type						
Clear-cell	22	18	28.1	4	10.8	0.101
Mucinous	51	26	40.6	25	67.6	
Endometrioid	24	17	26.6	7	18.9	
Serous	3	2	3.1	1	2.7	
Others	1	1	1.6	0	0.0	
CA125						
≤ 35 U/mL	48	24	13.0	24	23.8	0.008
> 35 U/mL	53	40	21.7	13	12.9	
Ascites volume						
≤ 100 mL	87	54	84.4	33	89.2	0.499
> 100 mL	14	10	15.6	4	10.8	
Ascites cytology						
Negative	94	58	90.6	36	97.3	0.203
Positive	7	6	9.4	1	2.7	

*CT* chemotherapy, *FIGO* International Federation of Gynecology and Obstetrics

### Oncologic outcome using original cohort

**Fig. 1:**
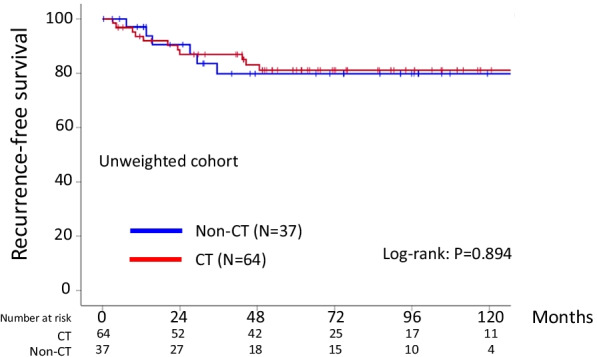
Kaplan–Meier-estimated recurrence-free survival (RFS) on stratifying by the presence or absence of chemotherapy {chemotherapy (N = 64) vs. observation (N = 37)}. The original cohort

On follow-up of all 101 women, 17 patients (16.8%) developed recurrence. Recurrence was observed in 11 (17.2%) in the chemotherapy group and 6 (16.2%) in the observation group (*P* = 0.900). Death was identified in 8 (12.5%) in the chemotherapy arm and 3 (8.1%) in the observation arm (*P* = 0.4864). The 5-year RFS rates of the chemotherapy and observation arms were 80.8 and 79.8%, respectively (Log-rank: *P* = 0.894: *N.S.*) (Fig. 1). Moreover, the 5-year OS rates of the chemotherapy and observation groups were 86.3 and 90.2%, respectively (Log-rank: *P* = 0.649: *N.S.*).

Subsequently, we conducted multivariate analyses to exclude selection bias from a variety of clinicopathologic covariates as strictly as possible. The age (> 35 years vs. ≤ 35 years), FIGO stage (IC2/IC3 vs. IA/IC1), histological type (non-mucinous vs. mucinous), preoperative CA125 value (> 35 vs. ≤ 35 U/mL), and performance of chemotherapy (no vs. yes) were employed in the Cox proportional multivariable analyses (Table 2). As the results, the FIGO stage was the only prognostic predictor for a shorter survival duration (RFS: *P* = 0.009/OS: *P* = 0.036). However, no association was identified between the presence of chemotherapy and a poorer oncologic outcome {RFS: HR (95% CI): 1.044 (0.168–1.742), *P* = 0.304/OS: HR (95% CI): 1.044 (0.230–4.732), *P* = 0.995}.

**Table 2:** Multivariate analyses in Cox hazard model (unweighted cohort)

Variable	Recurrence-free survival		Overall survival	
	HR (95% CI)	*P* value	HR (95% CI)	*P* value
Age		0.571		0.684
≤ 35 years	Referent		Referent	
> 35 years	0.710 (0.217–2.321)		0.744 (0.180–3.074)	
FIGO stage		0.009		0.036
IA/IC1	Referent		Referent	
IC2/IC3	4.229 (1.437–12.450)		4.048 (1.097–14.932)	
Histological type		0.918		0.859
Non-mucinous	Referent		Referent	
Mucinous	1.066 (0.317–3.579)		1.141 (0.268–4.860)	
CA125 value		0.253		0.991
≤ 35 U/mL	Referent		Referent	
> 35 U/mL	0.521 (0.171–1.592)		0.993 (0.271–3.633)	
Chemotherapy				
No	Referent	0.304	Referent	0.995
Yes	1.044 (0.168–1.742)		1.044 (0.230–4.732)	

*FIGO* International Federation of Gynecology and Obstetrics, *HR* hazard ratio

### Survival analyses in the weighted cohort

**Fig. 2:**
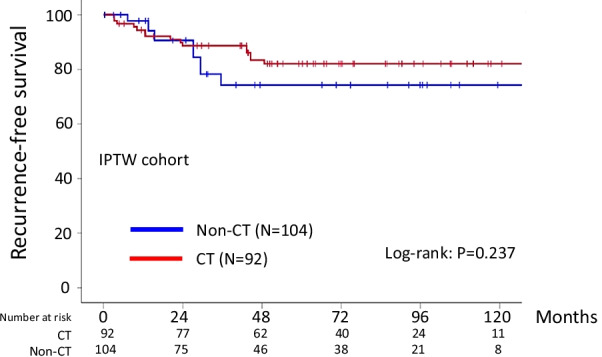
Kaplan–Meier-estimated recurrence-free survival (RFS) on stratifying by the presence or absence of chemotherapy {chemotherapy (N = 92) vs. observation (N = 104)}. The IPTW cohort

Calculation of PS for the presence or absence of chemotherapy was individually carried out using six clinicopathologic covariates: the age, FIGO substage, histological type, cytology of ascites, volume of ascites, and preoperative CA125 value. Consequently, 196 cases were generated using IPTW. Additional file 5: Table S3 summarizes clinicopathologic characteristics of the IPTW cohort. Following IPTW, all covariates except for the substage were well-balanced. Additional file 1: Figure S1 shows the distribution of PS before and after IPTW adjustment (Kernel density plots). After IPTW adjustment, the PS distributions of the two groups were equivalent, suggesting that backgrounds based on abovementioned confounders were appropriately balanced. In the IPTW cohort, the 5-year RFS (95% CI) rate was 82.1% for the chemotherapy arm and 74.3% for the observation arm (Log-rank: *P* = 0.237, *N.S.*: Fig. 2). Additionally, the 5-year OS rates were 87.0 and 90.5% in women with or without chemotherapy, respectively (Log-rank: *P* = 0.539, *N.S.*: Additional file 2: Figure S2).

**Fig. 3:**
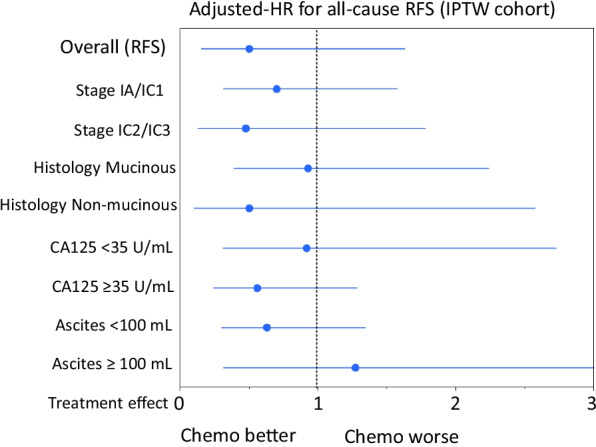
Forest plots for adjusted-HR for recurrence in the sub-groups of IPTW cohort {substage (IC2/IC3 vs. IA/IC1), histological type (mucinous vs. non-mucinous), preoperative CA125 value (≥ 35 vs. < 35 U/mL), and volume of ascites (≥ 100 vs. < 100 mL)}. Overall cohort: association of chemotherapy use and RFS was adjusted for clinicopathologic factors. Cox proportional hazard regression model using IPTW models. Circles represent adjusted-HR. Bars represent 95% confidence interval. HR, hazard ratio; RFS, recurrence-free survival; chemo, postoperative chemotherapy; and IPTW, inverse probability of treatment weighting

Also, in multivariable analysis, including the abovementioned confounders and PS, conducting chemotherapy was not a significant indicator of recurrence {adjusted-HR (95% CI): 0.501 (0.234–1.072), *P* = 0.075}. In multivariable analysis for OS, a similar trend was identified {adjusted-HR (95% CI): 0.939 (0.330–2.669), *P* = 0.905} (Table 3). Finally, we estimated the relative hazard of RFS for the performance of chemotherapy in IPTW-adjusted cohorts regarding substages (IC2/IC3 vs. IA/IC1), as well as the histological type (non-mucinous vs. mucinous), preoperative CA125 value (≥ 35 U/mL vs. < 35 U/mL), and volume of ascites (≥ 100 mL vs. < 100 mL). Figure 3 shows forest plots for the adjusted-HR for recurrence in the subgroups of IPTW cohort. Consequently, after the IPTW, the presence of chemotherapy was not a significant indicator of recurrence in all subgroups examined.

**Table 3:** Multivariable Cox proportional hazards analyses for RFS or OS among patients who had undergone FSS with or without adjuvant chemotherapy

	HR^#1^	95% CI	*P* value
*IPTW cohort*			
RFS			
Unadjusted	0.674	0.347–1.308	0.243
Adjusted for PS	0.651	0.335–1.265	0.205
Adjusted for PS, age, and sub-stage^#2^	0.534	0.268–1.065	0.075
Adjusted for PS and multi-factors^#3^	0.501	0.234–1.072	0.075
OS			
Unadjusted	1.327	0.534–3.202	0.542
Adjusted for PS	1.313	0.527–3.269	0.559
Adjusted for PS, age, and sub-stage^#2^	1.202	0.476–3.035	0.697
Adjusted for PS and multi-factors^#3^	0.939	0.330–2.669	0.905

*OS* overall survival, *FSS* fertility-sparing surgery, *IPTW* inverse treatment probability weighting, *PS* propensity score, *HR* hazard ratio, *95% CI* 95% confidence interval, #1: chemotherapy versus non-chemotherapy (referent), #2: IA/IB/IC1 versus IC2/IC3, #3: age (continuous), substage (IA/IB/IC1 vs. IC2/IC3), histological type (mucinous vs. non-mucinous), CA125 value (≤ 35 vs. > 35 U/mL), ascites cytology (positive vs. negative), and ascites volume (≤ 100 vs. > 100 mL)

## Discussion

In clinical practice, postoperative chemotherapy has been considered an effective strategy to ameliorate oncologic outcomes in patients with early-stage EOC. Our recent study demonstrated that, after adjustment for the clinicopathologic background, the performance of FSS did not reduce oncologic outcomes in such women, compared with conventional radical surgery [9]. One of the most critical questions was whether adjuvant chemotherapy use improves the survival of individuals with stage I EOC who have undergone FSS; in other words, whether chemotherapy itself can eliminate invisible metastasis of EOC. Indeed, the role of adjuvant chemotherapy for early-stage EOC has been debated for a long time. Here, to examine the effect of adjuvant chemotherapy in such women, we conducted a further multi-institutional study.

In the present study, we showed that the 5-year overall or recurrence-free survival rates in the two groups were 90.2/79.8 (chemotherapy group) and 86.3/80.8 (observation group), revealing no significant difference {Log-rank: *P* = 0. 649 (OS)/*P* = 0. 894 (RFS)}. Thus, confining to the current study, additional chemotherapy was not an independent prognostic factor in those patients. According to a combined analysis of two parallel randomized controlled trials collecting 925 women (477 in ICON1 [14] and 448 in ACTION [6]), the 5-year OS rate was significantly higher in the chemotherapy group than in the observation group {82 vs. 74%, respectively, *P* = 0.008} [15]. Likewise, a sophisticated meta-analysis including 1277 patients early-stage EOC also revealed increased RFS and OS rates with adjuvant chemotherapy { HR (95% CI): OS: 0.71 (0.53–0.93), RFS: 0.67 (0.53–0.84)} [16]. Nevertheless, one of the limitations regarding the evidence comes from the heterogeneity of the clinicopathologic background. Namely, a possible reason for the inconsistency is considered to be the difference in the distribution of age and histologic type. Actually, in the current study, the rate of mucinous/clear-cell histology, known to be common in women of reproductive age [3], was 72.3% (83/101). On the other hand, the same rate in the abovementioned parallel trials was only 33.5% (clear-cell/mucinous: 310/925). In fact, prior studies confirmed that clear-cell/mucinous carcinoma tends to show chemoresistance to taxane and/or platinum [17–19]. Indeed, according to previous studies, conducting chemotherapy did not necessarily lead to a survival advantage. Especially, in a retrospectively study by Nasioudis et al. including a total of 4811 patients with mucinous EOC, there was no difference in OS between patients who did (N = 1322) and did not (N = 2920) receive chemotherapy (5-year OS rate: 86.8 vs. 89.7%, respectively). Additionally, they also did not identify a difference following stratification by substage [20]. Similarly, based on a large-scale nationwide analysis recruiting 912 patients with stage IC mucinous EOC (chemotherapy use, n = 520 [57.0%]), adjuvant chemotherapy use was not associated with cause-specific survival {HR (95% CI): 1.296 (0.846–1.984), *P* = 0.233} [21]. Also, in stage I patients with clear-cell carcinoma, prior studies suggested that adjuvant chemotherapy had little impact on the survival benefit. Based on a retrospective study examining 219 patients with stage I clear-cell carcinoma {chemotherapy arm (N = 195), observation arm (N = 24)}, there were no significant differences in recurrence-free or overall survival between the two arms [22]. Regarding the chemotherapy cycles, Prendergast et al. reported that there was no impact of 3 vs. 6 cycles of chemotherapy on oncologic outcomes [23]. Although we could not come to a definite conclusion because this was a retrospective study, the effect of adjuvant chemotherapy in those women is considered to be limited.

In general, young patients with an encapsulated/well-differentiated EOC without extra-ovarian spread are appropriate candidates for FSS. However, we occasionally rencounter patients with IC/poor-differentiated EOC who strongly desire to preserve their fertility. In this study, we demonstrated that on IPTW, the RFS was equivalent between the two groups in the setting of substage (IA/IC1 vs. IC2/IC3) (Fig. 3). Consistently, according to nationwide retrospective analysis in stage I mucinous EOC, the IPTW-adjusted OS was equivalent between the chemotherapy and observation arms in the setting of capsule rupture alone (adjusted 4-year OS rate: 85.5 vs. 85.1%, respectively) as well as positive ascites cytology/surface involvement (adjusted 4-year OS rates 86.2 vs. 89.7%, respectively) [21]. We previously showed that young patients with stage IC2/3 EOC showed a poorer recurrence-free survival than those with stage IA or IC1 [24]. Indeed, intraperitoneal recurrences were more frequently observed in women with stage IC2/3 than IA/IC1 [25]. Therefore, we must keep in mind that patients with IC2/3 have a higher risk of recurrence, especially in the peritoneum, regardless of the performance of FSS or conventional surgery. Nevertheless, unfortunately, the current study included a limited number of patients with stage IC2/IC3 {14.9% (11/102)}. Moreover, to our knowledge, there is a limited number of reports focusing on the capsule status and analyzing oncologic outcomes with or without chemotherapy. In this context, we could not draw a clear conclusion with regard to the impact of chemotherapy on such women. Thus, we could not refute the utility of chemotherapy for such women when aiming to eliminate occult metastasis as thoroughly as possible.

In the present study, there were various limitations, reflecting the fact that many clinicopathologic factors were not as thoroughly controlled as they would be in a randomized controlled trial. Subsequently, some critical information was not provided, such as the socioeconomic status and detailed chemotherapy-related information, e.g., the dose duration and regimen change, which may have affected the reliability of the estimated propensity score. Thirdly, since patients enrolled in this study were part of a long-term multi-institutional study, the composition of the study subjects might have been influenced by many forms of bias. In particular, the majority of patients with IC2/IC3 tumors received chemotherapy. Thus, this study could not clarify the importance of chemotherapy for those patients. Lastly, the absence of a significant difference may be due to a lack of power reflecting the limited patient number. On the other hand, the strengths of this study included: firstly, the same chemotherapeutic protocol and criteria as for the identical study group; secondly, the practice of central pathological review by expert gynecologic pathologists; and lastly, the IPTW technique was utilized to adjust for different clinicopathologic covariates to minimize the weakness of the current retrospective study. Expectedly, women in the IPTW-adjusted dataset who received chemotherapy showed a generally equivalent survival trend, compared with those who did not undergo chemotherapy. Although it is difficult to come to a conclusion regarding the impact of chemotherapy, the current work may be useful for physicians and patients/families to share risk-and-benefit information before selecting chemotherapy. We need to reconfirm this in a future prospective study based on a larger population.

In conclusion, we investigated the question of how much adjuvant chemotherapy is associated with subsequent survival in young women with stage I EOC who had undergone FSS. We do not think that we had better change the present recommendation based on the current small-scale retrospective data. Actually, ovarian failure is often observed as a consequence of chemotherapy and is a major problem for women of reproductive age [26]. It is important for physicians and patients to estimate the risk-and-benefit of conducting chemotherapy in preoperative counseling, particularly patients without any sign of extra-ovarian spread. However, the present study had several limitations, including its non-prospective nature, various treatment modalities, heterogeneous substages, and possible lack of power. Additionally, as we diagnosed tumor recurrence based on findings in radiological images, there might be potential metastasis at any place, such as retroperitoneal lymph nodes. Furthermore, our current study could not clarify the importance of chemotherapy for patients with stage IC2/IC3 tumor. Thus, we do not refute the performance of chemotherapy may exert positive effect on the oncologic outcome, especially for women with high-risk factors, including positive ascites cytology and surface involvement. In this context, our current study is hypothesis-generating. The importance and significance of adjuvant chemotherapy for young women who have received FSS should be appropriately assessed in future prospective trials.

## Supplementary Information

**Additional file 1. Figure S1**: Frequency and Kernel density plots to depict the pre- (A) and post- (B) IPTW adjustment distribution of the propensity score in each treatment group.

**Additional file 2. Figure S2**: Kaplan–Meier-estimated overall survival (OS) on stratifying by the presence or absence of chemotherapy {chemotherapy (N = 92) vs. observation (N = 104)}. The IPTW cohort.

**Additional file 3. Table S1**: Distribution of the presence or absence of chemotherapy in each substage/histological type.

**Additional file 4. Table S2**: Independent predictors of adjuvant chemotherapy use for patients with stage I EOC who had received FSS.

**Additional file 5. Table S3**: Patients' characteristics (IPTW cohort).

## Data Availability

The data underlying this article cannot be shared publicly because of the privacy of individuals that participated in the study. The data will be shared on reasonable request to the corresponding author.
