# Supplementary figures and images for: Is adjuvant chemotherapy necessary for young women with early-stage epithelial ovarian cancer who have undergone fertility-sparing surgery?: a multicenter retrospective analysis
Source: BMC Womens Health. 2022 Mar 21;22:80. doi: 10.1186/s12905-022-01642-z (PMC8935788; doi:10.1186/s12905-022-01642-z)

## Slide 1
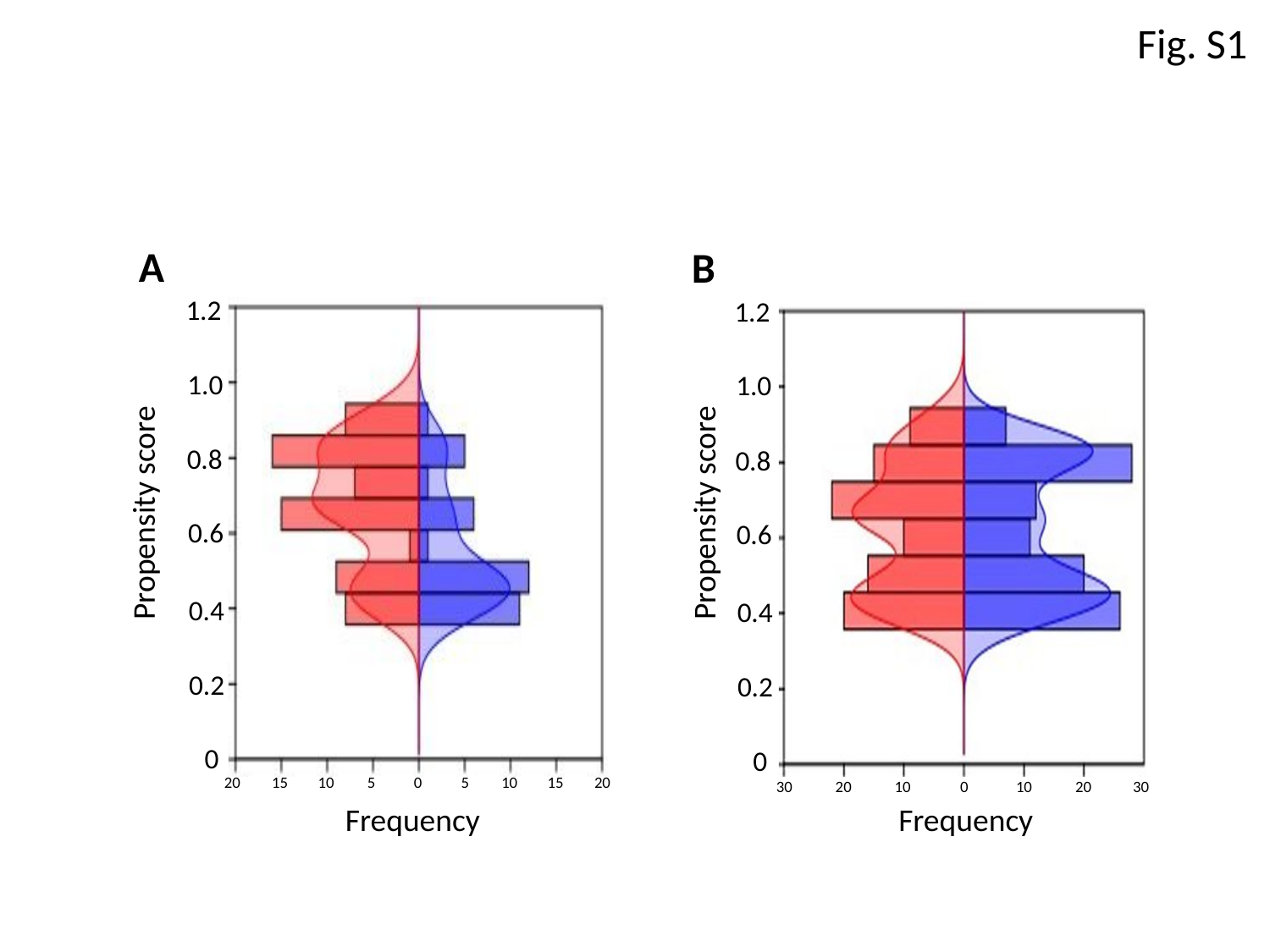

Fig. S1
A
B
1.2
1.2
1.0
1.0
0.8
0.8
Propensity score
Propensity score
0.6
0.6
0.4
0.4
0.2
0.2
0
0
20
15
10
5
0
5
10
15
20
30
20
10
0
10
20
30
Frequency
Frequency

Supplement: Supplementary file 1 — Additional file 1. Figure S1: Frequency and Kernel density plots to depict the pre- (A) and post- (B) IPTW adjustment distribution of the propensity score in each treatment group. [file 12905_2022_1642_MOESM1_ESM.pptx]
