# Supplementary material for: Is adjuvant chemotherapy necessary for young women with early-stage epithelial ovarian cancer who have undergone fertility-sparing surgery?: a multicenter retrospective analysis
Source: BMC Womens Health. 2022 Mar 21;22:80. doi: 10.1186/s12905-022-01642-z (PMC8935788; doi:10.1186/s12905-022-01642-z)
Supplement: Supplementary file 2 — Additional file 2. Figure S2: Kaplan–Meier-estimated overall survival (OS) on stratifying by the presence or absence of chemotherapy {chemotherapy (N = 92) vs. observation (N = 104)}. The IPTW cohort. [file 12905_2022_1642_MOESM2_ESM.pptx]

## Slide 1
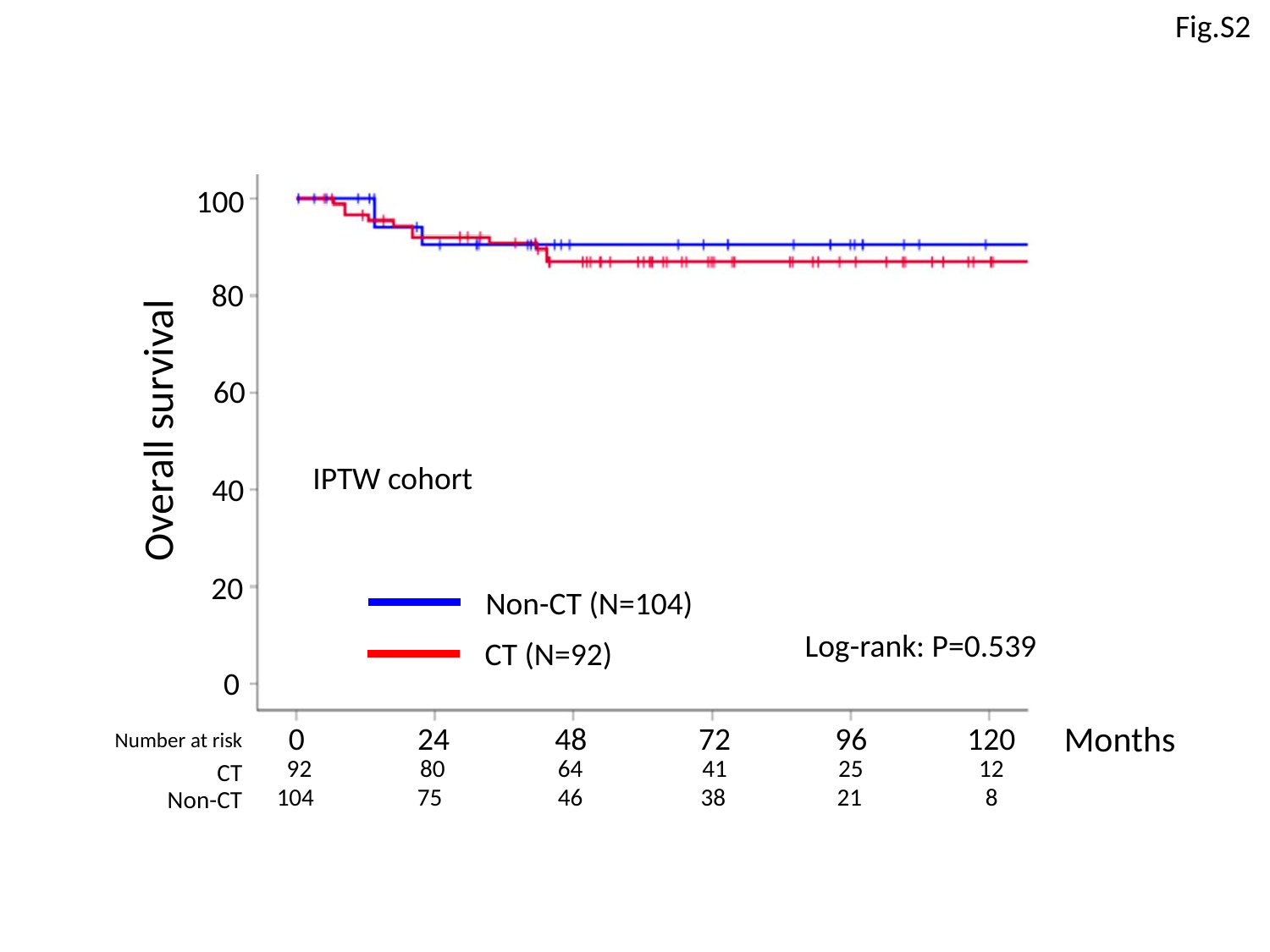

Fig.S2
100
80
60
Overall survival
Weighted cohort
IPTW cohort
40
Non-CT (N=104)
20
Non-CT (N=104)
Log-rank: P=0.539
CT (N=92)
Log-rank: P=0.539
CT (N=92)
0
Months
0
24
48
72
96
120
Number at risk
92
80
64
41
25
12
CT
104
75
46
38
21
8
Non-CT
