# Supplementary material for: Is adjuvant chemotherapy necessary for young women with early-stage epithelial ovarian cancer who have undergone fertility-sparing surgery?: a multicenter retrospective analysis
Source: BMC Womens Health. 2022 Mar 21;22:80. doi: 10.1186/s12905-022-01642-z (PMC8935788; doi:10.1186/s12905-022-01642-z)
Supplement: Supplementary file 3 — Additional file 3. Table S1: Distribution of the presence or absence of chemotherapy in each substage/histological type. [file 12905_2022_1642_MOESM3_ESM.docx]

| Table S1 |  |  |  |
| --- | --- | --- | --- |
| Distribution of the presence or absence of chemotherapy in each substage/histological type | | | |
| Chemotherapy: no/yes | IA-IB | IC1 | IC2-3 |
| CCC | 2/5 | 2/9 | 0/4 |
| non-CCC | 20/16 | 11/21 | 2/9 |
| CCC: clear-cell carcinoma |  |  |  |
