# Supplementary material for: Is adjuvant chemotherapy necessary for young women with early-stage epithelial ovarian cancer who have undergone fertility-sparing surgery?: a multicenter retrospective analysis
Source: BMC Womens Health. 2022 Mar 21;22:80. doi: 10.1186/s12905-022-01642-z (PMC8935788; doi:10.1186/s12905-022-01642-z)
Supplement: Supplementary file 4 — Additional file 4. Table S2: Independent predictors of adjuvant chemotherapy use for patients with stage I EOC who had received FSS. [file 12905_2022_1642_MOESM4_ESM.docx]

| Table S2 Independent predictors of adjuvant chemotherapy use for patients with stage I EOC who had received FSS. | | |
| --- | --- | --- |
| Variable | Odds ratio (95% CI) | *P*-value |
| **Age** |  | 0.602 |
| ≤35 years | Referent |  |
| >35 years | 1.282 (0.504-3.262) |  |
|  |  |  |
| **FIGO stage** |  | 0.049 |
| IA | Referent |  |
| IC | 2.462 (1.002-6.049) |  |
|  |  |  |
| **Histological type** |  | 0.322 |
| Non-CCC | Referent |  |
| CCC | 1.921 (0.528-6.981) |  |
|  |  |  |
| **CA125 value** |  | 0.030 |
| ≤35 U/mL | Referent |  |
| >35 U/mL | 0.362 (0.145-0.904) |  |
|  |  |  |
| **Ascites cytology** |  |  |
| Negative | Referent | 0.343 |
| Positive | 2.999 (0.310-29.050) |  |
| CT: chemotherapy, CCC: clear-cell carcinoma, FIGO: International Federation of Gynecology and Obstetrics | | |
