# Supplementary material for: Is adjuvant chemotherapy necessary for young women with early-stage epithelial ovarian cancer who have undergone fertility-sparing surgery?: a multicenter retrospective analysis
Source: BMC Womens Health. 2022 Mar 21;22:80. doi: 10.1186/s12905-022-01642-z (PMC8935788; doi:10.1186/s12905-022-01642-z)
Supplement: Supplementary file 5 — Additional file 5. Table S3: Patients' characteristics (IPTW cohort). [file 12905_2022_1642_MOESM5_ESM.docx]

| Table S3 Patients' characteristics (IPTW cohort) | | | | | | | |
| --- | --- | --- | --- | --- | --- | --- | --- |
|  |  | **CT** | |  | **Non-CT** | | *P*-value* |
|  | Total | N | % |  | N | % |  |
| **Total** | 196 | 92 |  |  | 104 |  |  |
| **Age** |  |  |  |  |  |  | 0.697 |
| ≤35 years | 120 | 55 | 59.8 |  | 65 | 62.5 |  |
| >35 years | 76 | 37 | 40.2 |  | 39 | 37.5 |  |
|  |  |  |  |  |  |  |  |
| **FIGO stage^#1^** |  |  |  |  |  |  |  |
| IA | 89 | 32 | 34.8 |  | 57 | 54.8 | 0.043 |
| IC1 | 82 | 47 | 51.1 |  | 35 | 33.7 |  |
| IC2 | 14 | 7 | 7.6 |  | 7 | 6.7 |  |
| IC3 | 11 | 6 | 6.5 |  | 5 | 4.8 |  |
|  |  |  |  |  |  |  |  |
| **Histological type** |  |  |  |  |  |  |  |
| Clear-cell | 44 | 21 | 22.8 |  | 23 | 22.1 | 0.193 |
| Mucinous | 104 | 42 | 45.7 |  | 62 | 59.6 |  |
| Endometrioid | 41 | 24 | 26.1 |  | 17 | 16.3 |  |
| Serous | 6 | 4 | 4.3 |  | 2 | 1.9 |  |
| Others | 1 | 1 | 1.1 |  | 0 | 0.0 |  |
|  |  |  |  |  |  |  |  |
| **CA125** |  |  |  |  |  |  |  |
| ≤35 U/mL | 97 | 46 | 50.0 |  | 51 | 49.0 | 0.893 |
| >35 U/mL | 99 | 46 | 50.0 |  | 53 | 51.0 |  |
|  |  |  |  |  |  |  |  |
| **Ascites volume** |  |  |  |  |  |  |  |
| ≤100 mL | 169 | 79 | 85.9 |  | 90 | 86.5 | 0.892 |
| >100 mL | 27 | 13 | 14.1 |  | 14 | 13.5 |  |
|  |  |  |  |  |  |  |  |
| **Ascites cytology** |  |  |  |  |  |  |  |
| Negative | 185 | 86 | 93.5 |  | 99 | 34.6 | 0.603 |
| Positive | 11 | 6 | 6.5 |  | 5 | 34.6 |  |
| CT: chemotherapy, FIGO: International Federation of Gynecology and Obstetrics, #1: FIGO 2014 | | | | | |  |  |
